# Sport and Disabled Individuals – Theory and Practice

**DOI:** 10.1515/hukin-2015-0085

**Published:** 2015-01-12

**Authors:** Anna Zwierzchowska, Krystyna Gawlik

**Affiliations:** 1 Special Physical Education Department, The Jerzy Kukuczka Academy of Physical Education in Katowice; 2 Posture Correction Department, The Jerzy Kukuczka Academy of Physical Education in Katowice

## Editors of Journal of Human Kinetics

On 26 June 2015 the 1st All-Poland Scientific Conference entitled **Sport and Disabled Individuals – Theory and Practice** took place at the Jerzy Kukuczka Academy of Physical Education in Katowice. The conference was held under the auspices of Professor Adam Zając, Rector of the Academy. We were able to bring together experts from numerous Polish higher education institutions as well as practitioners, trainers of paralympic teams, athletes, representatives of sports medicine and local media.

Apart from the scientific aspect, the aim of the event was to motivate the Polish academic community to engage in active promotion of sport for the disabled. The conference resulted in the exchange of information and experience. A lot of interesting observations were presented regarding the future of the Paralympics and in connection with the Act on Sport and its provision on Integration and Equalization of Opportunities for Persons with Disabilities. A moderated panel discussion on The Role of Science in Competitive Sport for the Disabled provided the basis for several conclusions that might help initiate or promote new strategies for the development of Paralympic sport and resulted in presenting the standpoint of an environment that is strongly interested in promoting dynamic development of competitive sport for the disabled. The discussion focused on two issues; first – devising systemic solutions at the level of research-study units, organizations, and sports associations for the disabled which would help trainers and competitors plan the training process (motor function tests, functional capacity evaluation, devising diets, supplementation, appropriate basis and modern training technologies). The experts believe this support system could only be implemented if its requirements (finances and staffing) will be divided between the Polish National Paralympic Committee, Sports Association for the Disabled “Start”, regional “Start” Associations and Academies of Physical Education. President of the the Polish National Paralympic Committee, Łukasz Szeliga – also attending the Conference, shares this opinion. Professor Stanis aw Kowalik stated that “...higher education institutions cannot and should not be service providers; they should function as a base which, due to broadening knowledge in the area, will contribute to the development of new ideas”. They should carry out research for the needs of sports practice. In practice this would mean sports medicine specialists, physiologists and psychologists planning and carrying out diagnostic procedures to monitor the competitors’ health condition. The resultant data would provide material for scientific research performed to enrich theoretical knowledge and optimize training in different sport disciplines. All this might, in turn, contribute to the development of a support system for training persons with disabilities.

Scientific exploration and identification of disabled athletes’ problems will broaden our knowledge on the mechanisms and character of their training and thus will also help improve training programs and prognosis of training outcomes. Scientific exploration and creation/verification of new ideas will undoubtedly make a valuable contribution to the training process of disabled athletes. The quality of education and preparation needed to become an athletic trainer for the disabled will also improve, which is important as – according to Ryszard Plinta, coach of the national Paralympic rugby team and associate professor of the Medical University of Silesia – “guidelines and good practices are still lacking in numerous sport disciplines practised by the disabled, and trainers must frequently act by intuition”. Considering current knowledge, technological progress and modern-day potential of medical science, education of athletic trainers should be based on multidisciplinary knowledge and achievements. If we aim to encourage competitive sport among the disabled, they must be provided with the feeling of maximum health safety. In his presentation, Wojciech Gawroński, MD PhD, the head of the Polish sports medicine team at Paralympics, emphasized progress in sports medicine and the importance of prophylactic examinations as the key to sporting career and success. It is only when the disabled can be sure their health is not put to risk that more and more of them will engage in competitive sport.

The other focus of panel discussion concerned the promotion of paralympic sports among the disabled and able-bodied people. Encouraging sports participation among the disabled and making them feel they might achieve well-being and fulfilment through sport should go along with making the able-bodied members of the society aware of the fact that disabled people can reach their full potential. It is essential to equally depict Paralympic and Olympic events. Hence the important role of the media although experts in the field believe that those concerned should become more extroverted.

Editor Marianna Dufek-Durczok pointed out that grassroot solutions are also important and agreed with Professor Andrzej Kosmol, who said that “... disability sport should draw attention to athletes’ skills and not limitations”. Thus, there is a need to overcome the stereotype of a disabled person and get used to seeing athletes with disabilities. Integrating disabled children into regular schools may bring considerable benefits since being in a classroom with disabled students helps realize their potential. Hence, higher education institutions, including Academies of Physical Education and Medical Universities should encourage voluntary services of their students at disability sports events.

We hope that this conference will not only become a permanent component of the Academy’s activities but will also markedly contribute to the campaign of Silesian higher education institutions for the promotion and improved quality of sport for the disabled.

